# Enhancing osseointegration in dental implants with topical platelet - rich fibrin

**DOI:** 10.6026/9732063002001560

**Published:** 2024-11-30

**Authors:** Mohammed Minhaj Ahmed Amer, Amit Kumar Sharma, Mahendra Azad, Nisha Sharma

**Affiliations:** 1 Department of Oral and Maxillofacial surgery, NIMS University, Jaipur, Rajasthan, India; 2 Honoary Clinical Director, Clove Dental, India; 3 Department of Neuro Anesthesiology, Mahatma Gandhi Medical College and Hospital, Jaipur, Rajasthan, India

**Keywords:** Dental implants, platelet-rich fibrin, resonance frequency analysis, torque

## Abstract

The impact of platelet-rich fibrin as a topical application on osseointegration in dental implants is of interest. Hence, a medical questionnaire was used to gather information during the initial interview, including details about past and current medical conditions, temporary and chronic medications, smoking habits and any special dietary restrictions. To assess primary stability, RFA values were recorded on the day of implant placement using the Ostell instrument. A highly significant difference was observed between RFA1 and RFA2 in the buccolingual direction (p ≤ 0.05). The topical application of PRF on implants led to a significant improvement in osseointegration around dental implants compared to conventionally placed implants.

## Background:

Oral implants provide an effective means of restoring natural dentition, thereby enhancing contour, functionality, comfort, aesthetics, speech and overall oral health [1]. A fundamental component for the success of dental implants is osseointegration, which involves the direct structural and functional connection between living bone and the implant surface, a concept first histologically described by Branemark in 1983 [1]. Extensive research has been conducted to improve the quality of osseointegration and expedite its process since Branemark's initial findings. Osseo integration is a therapeutic process that is reliant on achieving implant stability, which can be divided into primary and secondary stability phases [2]. Primary stability occurs immediately after implant placement and is influenced by factors such as bone density, mechanical attributes, implant design, edentulous zone challenges and surgical technique [2]. Secondary stability involves several contributing factors, including the initial implant stability, the surgical technique used, the condition and volume of the bone, the incision healing, implant design and coating, implant length, masticatory forces and prosthetic design [3]. A notable drawback of implants is the osseointegration waiting period, typically lasting 5-6 months in the maxilla and 3-4 months in the mandible [4]. Premature loading during this healing phase can significantly compromise implant longevity, causing discomfort and functional limitations for patients. Numerous strategies have been explored to enhance and accelerate the osseointegration process in dental implants [4-5]. These strategies include the use of various implant designs and surface treatments, incorporation of plasma rich protein, utilization of stem cells and adjunctive therapies such as pulsed electromagnetic field (PEMF), low-level laser therapy (LLLT) and low-intensity pulsed ultrasound (LIPUS) [6, 7-8]. Injectable platelet-rich fibrin (I-PRF) is a third-generation platelet concentrate shown to have beneficial effects on fibroblasts and osteoblasts, influencing the release of different growth factors vital for healing and vascular development [9, 10]. It promotes faster wound healing, vascularization, cost efficiency and immune compatibility. Despite these benefits, research on the effects of platelet-rich fibrin as a surface treatment on osseointegration in dental implants remains limited. Therefore, it is of interest to describe this study which aims to assess the impact of topical application of platelet-rich fibrin on osseointegration in dental implants, with implant stability being measured using Resonance Frequency Analysis (RFA).

## Materials and Methods:

## Patient selection and consent:

Patients were selected and their treatments were administered following the provision of informed consent. The study protocol was sanctioned by the ethical committee of the NIMS Dental College & Hospital.

## Data collection:

A comprehensive medical questionnaire was utilized, which encompassed data from the initial interview, historical and current medical conditions, medications (both chronic and temporary), smoking habits and special dietary restrictions. Additionally, the questionnaire included specific inquiries regarding the quality of any current prosthesis, if applicable and causes of tooth loss, categorized into caries, periodontal disease, trauma, or other factors.

## Inclusion criteria:

[1] Patients who provided informed consent.

[2] Individuals aged 18 years and older.

[3] Patients maintaining excellent oral hygiene standards.

[4] Individuals requiring two or more dental implants due to missing teeth.

[5] Sufficient hard tissue available at the implantation site to support implant procedure.

## Exclusion criteria:

[1] Presence of active periodontal disease.

[2] Chronic smoking or tobacco chewing habits.

[3] Uncontrolled diabetes.

## Clinical examination and diagnostics:

Vital signs, including blood pressure, temperature, respiratory rate and pulse rate, were assessed for all participants. Hematological examinations conducted on all ten patients included complete blood profile (CBP), clotting time (CT), bleeding time (BT), platelet count, HIV and Hepatitis-B screening, serum calcium level and blood glucose tests. The intraoral examinations were thorough, involving assessments of oral hygiene, periodontal health, ridge defects, arch relationships, inter-foraminal spacing, mucosal quality and quantity, bone contour and inter-/intra-maxillary relationships, including available inter-arch distance documentation.

## Implantation procedure:

In twelve patients, a total of twenty-four implants were placed. Resonance Frequency Analysis (RFA) values were documented on the day of implant placement to assess primary stability, utilizing the Ostell device. All patients underwent identical procedures for stability testing. Orthopantomograms (OPG) were acquired immediately post-placement. Secondary stability of the implants was assessed after a 90-day interval, again using the Ostell instrument.

## Statistical analysis:

Data recorded was compiled and input into Microsoft Excel 2019, followed by exportation to the data editor page of SPSS version 15 (SPSS Inc., Chicago, Illinois, USA). Quantitative variables were depicted using means and standard deviations or medians and interquartile ranges, contingent upon their distribution. Qualitative variables were presented as numbers and percentages. A confidence level of 95% and a significance level of 5% were maintained for all statistical tests.

## Results:

In the test group, 33.3% of the subjects had bone type D1, while 66.7% had bone type D2. Similarly, in the control group, 33.3% of the subjects exhibited D1 bone type and 66.7% exhibited D2, as illustrated in Table 1 and Graph 1. Therefore, there was no significant difference in bone quality between the experimental and control groups (p=1.0). In the test group, the mean initial insertion torque (IIT) was 42.08 ± 3.343, while in the control group, it was 40.83 ± 1.030 (Table 2) (Graph 2). No significant difference in the mean IIT between the two study groups was detected by the independent samples t-test (p=0.229). The mean and standard deviation for the first RFA in the mesiolingual direction for the test group is 64.33 ± 6.18, while the second RFA for the test group is 74.17 ± 5.357. In the control group, the mean for the first RFA is 62 ± 5.560 and the mean for the second RFA is 66 ± 3.790. In the test group, the mean RFA1 measurement in the mesiodistal direction was 64.33 ± 6.184, while the mean RFA2 measurement was 74.17 ± 5.357. A highly significant difference between RFA1 and RFA2 in the mesiodistal direction was identified (p=0.000) using the paired-samples t-test. For the buccolingual direction, the mean RFA1 value was 65.17 ± 5.323 and the mean RFA2 was 74.75 ± 5.429. A highly significant difference was also found between RFA1 and RFA2 in the buccolingual direction (p=0.000), as determined by the paired-samples t-test (Table 2). In the control group, the mean RFA1 measurement was 62.0 ± 5.56, while the mean RFA2 measurement in the mesiodistal direction was 66.0 ± 3.79. The paired-samples t-test revealed a highly significant difference between RFA1 and RFA2 in the mesiodistal direction (p=0.008). For the buccolingual direction, the mean RFA1 was 62.92 ± 6.598 and the mean RFA2 was 67.67 ± 3.75. Similarly, a highly significant difference was found between RFA1 and RFA2 in the buccolingual direction (p=0.008), according to the paired-samples t-test (Table 3). The values for Pearson's correlation coefficient are presented in Table 4. No significant correlation was observed between bone quality and IIT or between bone quality and RFA1. However, there is a significant positive correlation between IIT and RFA1 (r=0.468, p=0.021).

## Discussion:

Recent research has focused on enhancing osseointegration, which can be categorized into two main groups: techniques that improve the biocompatibility of the implant surface and those that augment the tissue response. The factors influencing osseointegration can be classified into several categories: those related to the implant, the condition of the host bone bed, mechanical stability, the use of adjuvant therapies and the remodelling of periprosthetic bone at the interface. This in vivo study aims to assess the impact of Platelet-Rich Fibrin (PRF) on bone healing around endosseous implants utilizing Resonance Frequency Analysis (RFA) with the Ostell device. The Touareg TM-S Spiral implant, known for its unique tapered spiral design, was employed in this study, recognized as an effective configuration for both immediate and delayed placements. Self-tapping implants were chosen to ensure optimal primary stability, which has long been regarded as critical for implant success [9, 10- 11]. Through RFA, clinicians can assess the stability of implants immediately following placement and throughout the healing process [12, 13]. This aids in determining whether additional healing time is required before securing the prosthetic tooth and helps identify patients at risk due to compromised bone tissue or other factors [13]. In the current study, no statistically significant differences were observed in bone quality between the test and control groups (p=1.0). The mean initial insertion torque (IIT) recorded in the test group was 42.08 ± 3.343, while the control group exhibited a mean IIT of 40.83 ± 1.030, with no significant difference noted (p=0.229). These findings align with those reported by Öncü E and Alaaddinoglu, which similarly indicated no significant differences in insertion torque values (P = .632) [14]. Furthermore, RFA1 measurements exhibited no significant difference between the test and control groups, both in the mesiodistal (MD) direction (p=0.342) and the buccolingual (BL) direction (p=0.368). This is consistent with Öncü *et al.*'s findings, which indicated that PRF application significantly enhanced implant stability during the initial healing phase, as reflected by higher Implant Stability Quotient (ISQ) values [14]. Similarly, Torkzaban *et al.* evaluated the effect of PRF on dental implant stability, concluding that its application at the osteotomy site could prevent or mitigate early decreases in implant stability and promote osseointegration [15]. In the test group, a paired-samples t-test revealed a significant increase between RFA1 and RFA2 measurements in both the mesiodistal (p=0.000) and buccolingual (p=0.000) directions, indicating that the application of i-PRF may enhance osseointegration due to its growth factor content. These results are in line with Tabrizi *et al.* who assessed implant stability in the posterior maxilla with and without PRF throughout the healing period, noting that both groups exhibited substantial secondary stability, although the test group demonstrated higher RFA2 values [16]. The study also found no statistically significant correlation between bone quality and torque, nor between bone quality and RFA1; however, a small sample size allowed for the identification of a statistically significant positive correlation between torque and RFA1. Limitations of this study include a small sample size and a brief follow-up period. Future investigations into the use of i-PRF in implant dentistry are warranted, particularly given its recent introduction and this study exclusively involved implants placed in D1 and D2 bone, excluding D3 and D4 classifications.

## Conclusion:

The topical application of PRF on implants resulted in a significant improvement in osseointegration compared to conventionally placed implants, no statistically significant correlation was found between bone quality and torque, no statistically significant correlation was noted between bone quality and RFA1 and a statistically significant positive correlation was identified between torque and RFA1.

## Figures and Tables

**Table 1:** Distribution of the study subjects based on the quality of bone in the test and control groups

**Variables**			**Groups**		**Total**	
			**Test group**	**Control group**		
	D1	Count	4	4	8	
		% within bone quality	50.00%	50.00%	100.00%	
Bone quality		% within group	33.30%	33.30%	33.30%	
	D2	Count	8	8	16	
		% within bone quality	50.00%	50.00%	100.00%	
		% within group	66.70%	66.70%	66.70%	
Total		Count	12	12	24	
		% within bone quality	50.00%	50.00%	100.00%	
		% within group	100.00%	100.00%	100.00%	

**Table 2:** Mean difference between the RFA1 and RFA2 values in the test group in the mesiodistal and buccolingual directions

**Variables**	**Paired Differences**					**Sig. (2-tailed)**
	**Mean difference**	**Std. Deviation**	**Std. Error Mean**	**95% Confidence Interval of the Difference**		
				**Lower**	**Upper**	
Mesiodistal	-9.833	4.196	1.211	-12.499	-7.167	0.000*
Buccolingual	-9.583	4.889	1.411	-12.69	-6.477	0.000*

**Table 3:** Mean difference between the RFA1 and RFA2 values in the control group's mesiodistal and buccolingual directions

	**Mean**	**Std. Deviation**	**Std. Error Mean**	**95% Confidence Interval of the Difference**		
				**Lower**	**Upper**	
Mesiodistal	4	4.264	1.231	-6.709	-1.291	0.008*
Buccolingual	4.75	5.119	1.478	-8.002	-1.498	0.008*

**Table 4:** Correlation between the quality of bone, IIT and RFA1

**Variables**	**Test utilized**	**Quality of bone**	**IIT**	**RFA1**
Quality of bone	Pearson Correlation	1	-0.092	-0.109
	Sig. (2-tailed)		0.669	0.461
	N	48	24	48
IIT	Pearson Correlation	-0.092	1	0.468
	Sig. (2-tailed)	0.669		.021*
	N	24	24	24
RFA1	Pearson Correlation	-0.109	0.468	1
	Sig. (2-tailed)	0.461	.021*	
	N	48	24	48
